# Preparation of a novel fracturing fluid with good heat and shear resistance†

**DOI:** 10.1039/c8ra09483g

**Published:** 2019-01-09

**Authors:** Yang Zhang, Jincheng Mao, Tao Xu, Zhaoyang Zhang, Bo Yang, Jinhua Mao, Xiaojiang Yang

**Affiliations:** State Key Laboratory of Oil & Gas Reservoir Geology and Exploitation, Southwest Petroleum University Chengdu Sichuan China jcmao@swpu.edu.cn 790016605@qq.com 815675989@qq.com

## Abstract

A new terpolymer (MAS-1) was created by copolymerizing acrylamide (AM), acrylic acid (AA), and 4-isopropenylcarbamoyl-benzene sulfonic acid (AMBS). Thermogravimetric analysis (TGA) suggested that MAS-1 has better heat resistance and meets the requirements for a fracturing fluid at 200 °C. X-ray diffraction (XRD), and conductivity tests showed that MAS-1 has good water solubility. The viscosity of the fracturing fluids containing MAS-1 was found to be about 135 mPa s after 120 min at 170 s^−1^ and 150 °C. SEM images and the determination of viscoelasticity showed that MAS-1 has a dense and robust spatial network structure in the fracturing fluid. The sedimentation velocity of the proppant was 0.0528 cm min^−1^ at 90 °C. When enough ammonium persulfate was added to yield approximately 0.10 wt%, the viscosity of the broken fluid was 4.5 mPa s, and the gel broken fluid was transparent without visible residue. In addition, the fracturing fluid did little damage to the reservoir. The drag reduction rate of MAS-1 was always higher than KYPAM-6A and HPAM with the shear rate ranging from 1000 s^−1^ to 7000 s^−1^. Therefore, this fracturing fluid could be an alternative for low permeability reservoir stimulation.

## Abstract 2

A new terpolymer (MAS-1) was created by copolymerizing acrylamide (AM), acrylic acid (AA), and 4-isopropenylcarbamoyl-benzene sulfonic acid (AMBS).

## Graphical abstract

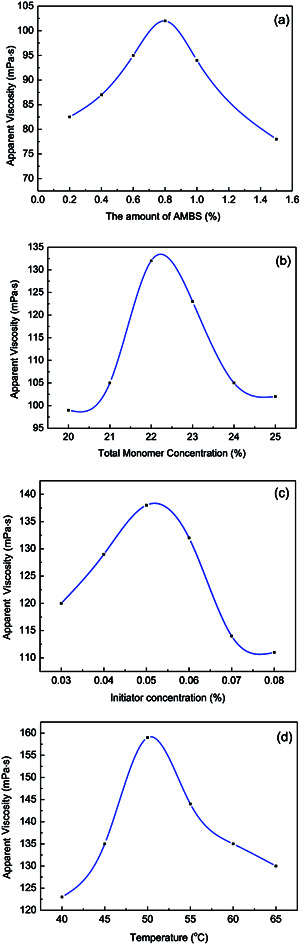

## Introduction

With the development of the oil and gas industry, low-permeability reservoirs account for an increasing proportion of the total oil and gas resources.^1–3^ In order to successfully exploit these resources, reservoir stimulation must be carried out. Hydraulic fracturing is a technique widely adopted to stimulate the production of low-permeability oil and gas wells.^4–7^ It has been used in well stimulation for more than 40 years.^8^ As an indispensable component of fracking, fracturing fluid ultimately affects the success or failure of fracking.^9,10^ In the last decade, domestic and foreign scholars have conducted extensive research on fracturing fluids. Various fracturing fluid systems have been investigated, such as guar gum fracturing fluid, polymer fracturing fluid, foam fracturing fluid, acid-based fracturing fluid, emulsified fracturing fluid, oil-based fracturing fluid and VES fracturing fluid.^11^ Meanwhile, because of their low frictional resistance, good sand-suspension, and lower filter loss, guar gum and polymer-based fracturing fluids are the main fracturing fluids used in oilfields.^12–14^

However, from a field operational point of view, guar gum-based fracturing fluids usually have the disadvantages of high insoluble residue, poor shear resistance, and large damage to reservoirs due to the plugging of pore throats. Hence, it difficult to use guar gum fracturing fluids in high-temperature and low-permeability oil and gas wells.^15^ Not only that, because guar gum is mainly imported from places such as Pakistan, India, and Brazil, the availability and price of guar gum is subject to fluctuations. Moreover, synthetic polymers have the advantages of strong thickening ability, good gel stability, strong ability to carry sand, insensitivity to bacteria, less residue formation, and low damage to reservoirs.^15–18^ Nevertheless, polymer-based fracturing fluids are unstable at high temperatures and high shear rates. In addition, the water solubility of synthetic polymers is poor compared to viscoelastic surfactants. Based on the early work with fracturing fluids, it was believed that the heat-resistance and shear-resistance of polymers could be improved by introducing a benzene ring, and the water solubility of polymers could be improved by introducing strongly hydrophilic groups, such as sulfonate and carboxyl groups. Naturally, the terpolymer MAS-1 was synthesized by copolymerizing acrylamide (AM), acrylic acid (AA), and 4-isopropenylcarbamoyl-benzene sulfonic acid (AMBS). The benzene ring improves the rigidity of the polymer, which will keep the main chain of the polymer stretched in solution, giving the polymer solution an excellent resistance to temperature and shear.

In addition, organic zirconium is used to crosslink with the polymer. This further improves the temperature and shear resistance of the fracturing fluid, allowing the fracturing fluid to transport the proppant deeply into the cracks. Using MAS-1 in fracturing fluids could improve the stimulation efficiency of fracking, which could greatly increase the production of oil and gas wells. Therefore, it is important to investigate this fracturing fluid as an alternative for low-permeability reservoir stimulation.

## Experimental

### Materials

Acrylamide (AM), acrylic acid (AA), sodium hydroxide, 2,2′-azobis (2-methylpropionamide) dihydrochloride (V50), ammonium persulfate and HPAM (*M*_w_ = 16–18 × 10^6^) were purchased from the chemical market. KYPAM-6A having 26.4% degree of hydrolysis, *M*_w_ = 25.14 × 10^6^, was purchased from the Hengju Oil Field Chemical Reagents Co., Ltd. (Beijing, China). 4-Isopropenylcarbamoyl-benzene sulfonic acid (AMBS) is a functional monomer prepared in our lab. Zirconium crosslinking agent (Zr-CL) was prepared in our lab by chelating zirconium oxychloride octahydrate with triethanolamine and glycerol at a certain temperature. All chemicals were of analytical reagent grade and were utilized without further purification.

### Measurements and fluid preparation

#### (i) Fluid preparation

First, 997 g of deionized water in the beaker was stirred on the agitator. When a vortex is formed in the water, 3 g of terpolymer MAS-1 was added into the water slowly to prevent the formation of insoluble particles. After the terpolymer was completely dissolved in water, it was kept at 25 °C for 24 h before being used. In other words, the 0.3 wt% polymer aqueous solution was obtained.

#### (ii) Shear resistance

50 mL of the above 0.3 wt% polymer aqueous solution was taken in a measuring cylinder and was placed into the RS600 rheometer. Then, the polymer aqueous solution was subjected to continuous shear testing with a shear rate ranging from 7.34 s^−1^ to 1000 s^−1^ under 25 °C for 30 min using a HAAKE RS600 rheometer. The viscosity retention rate was calculated by the viscosity at 1000 s^−1^ divided by the viscosity at 10 s^−1^.

#### (iii) Rheological property measurement

The fracturing fluid was prepared by mixing 0.3 wt% MAS-1 aqueous solution and 0.8 wt% Zr-CL. First, the fracturing fluid was heated from 25 °C to 150 °C with a step length of 10 °C under the shear rate of 170 s^−1^ in the HAAKE RS600 rheometer. Then, the rheology of fracturing fluid was measured at the temperature of 150 °C and the shear rate of 170 s^−1^ for 110 min using a HAAKE RS600 rheometer.

The detailed experimental procedures can be found in the ESI.†

## Results and discussion

### Synthesis of MAS-1

**Scheme 1:**
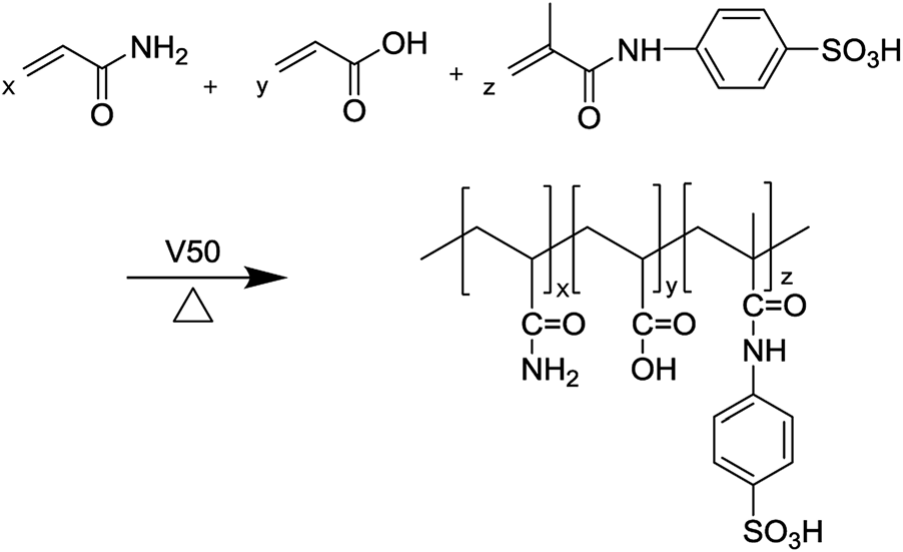
Synthesis of MAS-1.

Certain proportions of acrylamide (AM), acrylic acid (AA), and 4-isopropenylcarbamoyl-benzene sulfonic acid (AMBS) were added into a 100 mL beaker in deionized water under an inert nitrogen atmosphere. The pH value was adjusted to 7.0 by sodium hydroxide. Copolymerization was allowed to be initiated by a certain amount of V50 at 50 °C for 4 h. Then, the polymer was obtained and cut into small pieces, which were dried at 50 °C under a vacuum desiccator for approximately 10 h after being purified repeatedly with ethanol, and named it as MAS-1. The synthesis process is shown in Scheme 1.

### Optimal synthesis conditions of MAS-1

**Fig. 1:**
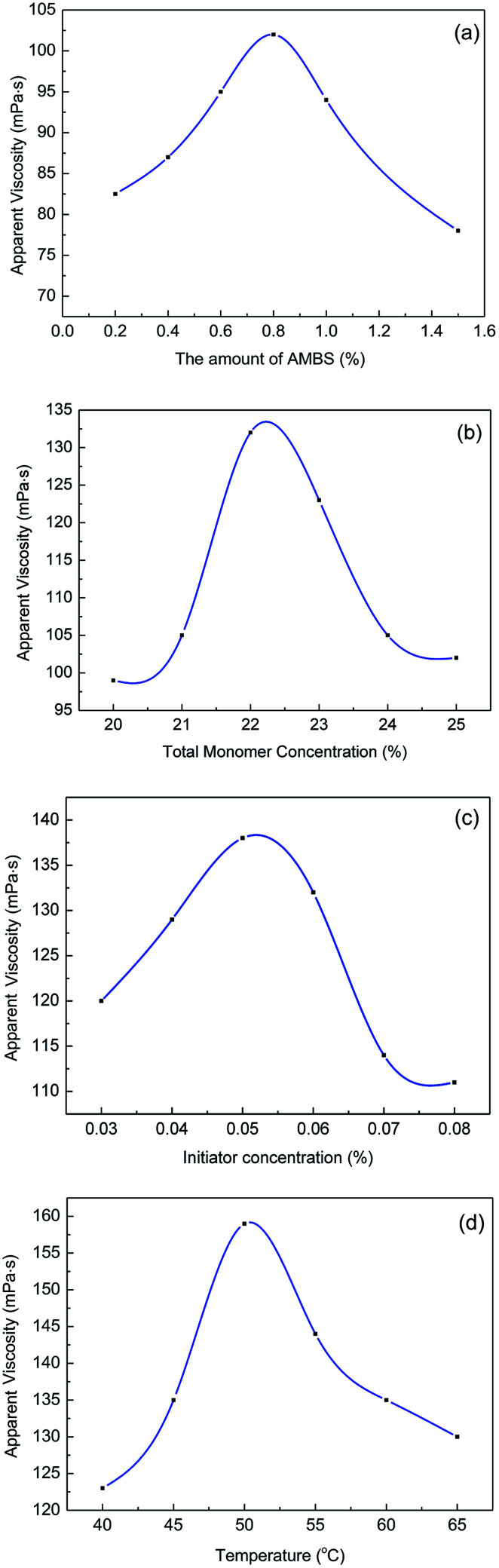
Effects of synthetic factors on the solution viscosity of MAS-1.

The variation in the apparent viscosity of 0.3 wt% copolymer solutions was determined by a single variable method with different synthetic conditions. All these experiments are based on a neutral pH solution, a polymerization time of 4 h, and an acrylamide and acrylic acid molar ratio of 80 : 20. The viscosities were measured with a ZNN-D6 viscometer at room temperature (25 °C). The experimental results were shown in Fig. 1.

Fig. 1(a) shows that increasing the amount of AMBS increased the viscosity of the MAS-1 solutions at first, followed by decreasing viscosity. We concluded that adding AMBS appropriately enhanced the polymer rigidity and the ability to form hydrogen bonds within and between the molecules. However, once the amount of AMBS was higher than 0.8 mol%, the steric effect of AMBS slowed the polymerization, resulting in a decrease in molecular weight and viscosity.

Fig. 1(b) shows that the maximum viscosity was reached with a 22 wt% total monomer concentration. We concluded that when the total monomer concentration was less than 22 wt%, the probability of collision among monomers was lower, leading to slower polymerization and the faster termination of free radicals, causing the molecular weights of the polymers to be lower. When the monomer concentration exceeded 22 wt%, the increase in collision frequency released a lot of heat that could accelerate the rate of polymerization, chain transfer, and chain termination. These reactions will reduce the molecular weight of the copolymer, which will lead the viscosity of the copolymer solutions to decrease.

Fig. 1(c) shows that increasing the amount of initiator led to an initial increase in the viscosity of MAS-1 solutions, followed by a decrease. We concluded that initially increasing the amount of initiator led to more free radicals, causing a greater conversion rate of the monomers while also causing the viscosity of the MAS-1 solution to increase. However, when the concentration of the initiator was too high, an excessive number of free radicals were generated by the decomposition of the initiator, which also accelerated the rate of polymerization, chain transfer, and chain termination. Meanwhile, the temperature of the gel rose sharply, causing an implosion of the gel and decreasing the apparent viscosity of the copolymer solution.

Fig. 1(d) shows an inverted V-shaped curve between the viscosity and temperature. We concluded that the rate of production of free radicals was inhibited by the low temperature. Increasing the temperature increased the number of free radicals, which also increased the rate of reaction and accelerated the rate of chain termination. However, at temperatures higher than 50 °C, many free radicals were produced during a very short time, resulting in a lower chain propagation rate, and the molecular weight of the final product decreased.

Therefore, we concluded that the optimum synthesis conditions for MAS-1 were (1) a total monomer concentration of 22 wt%, (2) a pH of 7.0 for the solution, (3) an initiator concentration of 0.05% of the mass of the total monomers, (4) a reaction temperature of 50 °C, (5) an AM to AA mole ratio of 80 : 20, and (6) an AMBS concentration of 0.8% of the moles of the total monomers.

### Characterization

#### (i) IR analysis

**Fig. 2:**
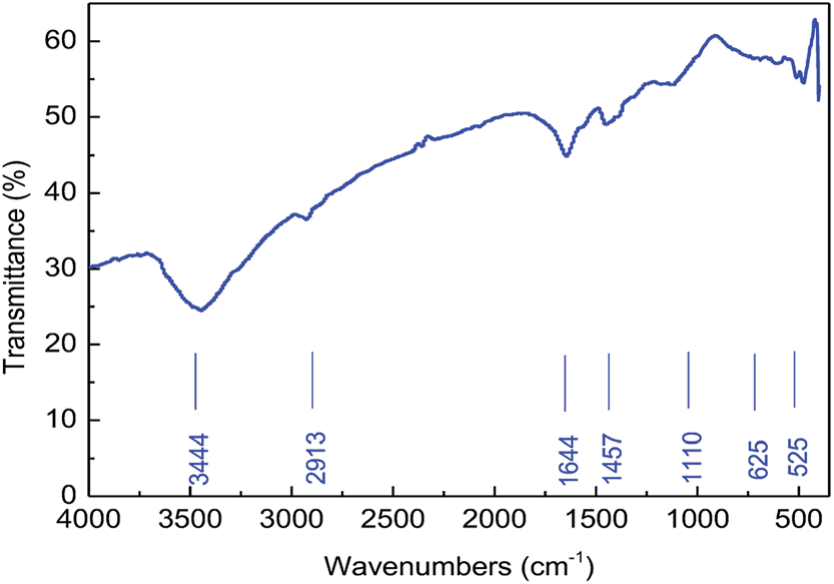
The FT-IR spectrum of MAS-1.

The structure of MAS-1 was confirmed by FT-IR. Fig. 2 shows the FT-IR spectra of MAS-1. The peak at 3444 cm^−1^ is due to –N–H. The peak at 2913 cm^−1^ is generated by the antisymmetric vibration of C–H. The strong absorption peak at 1644 cm^−1^ is created by the stretching vibration of –C=O in the amide group. The peak at 1110 cm^−1^ is due to the stretching vibration of –C–N–. The peak at 1110 cm^−1^ comes from the stretching vibration of –C–C–. In addition, the absorption peaks at around 625 cm^−1^ and 525 cm^−1^ in the IR spectrum were due to the stretching vibrations of the –SO_3_^2−^ groups. The results verified that the synthesized polymer was consistent with the designed MAS-1.

#### (ii) ^1^H NMR analysis

**Fig. 3:**
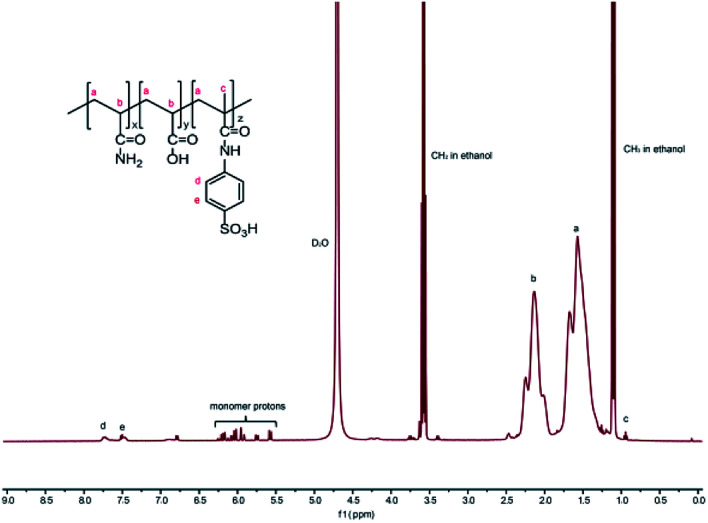
The ^1^H NMR spectrum of MAS-1.

The resonances of protons are as follows: 4.70 (D_2_O) for the solvent peak, 1.57–1.68 ppm (a) and 2.02–2.25 ppm (b) for (–CH_2_–CH–), 0.93–0.96 ppm (c) for the methyl proton peak of AMBS, 7.47–7.52 ppm (e) and 7.70–7.75 ppm (d) for the benzene ring proton of AMBS, 1.09–1.12 ppm and 3.55–3.63 ppm for the ethanol solvent peaks. It was verified that the synthesized polymer was consistent with the designed MAS-1 (Fig. 3).

### Thermogravimetric analysis (TGA) of MAS-1

**Fig. 4:**
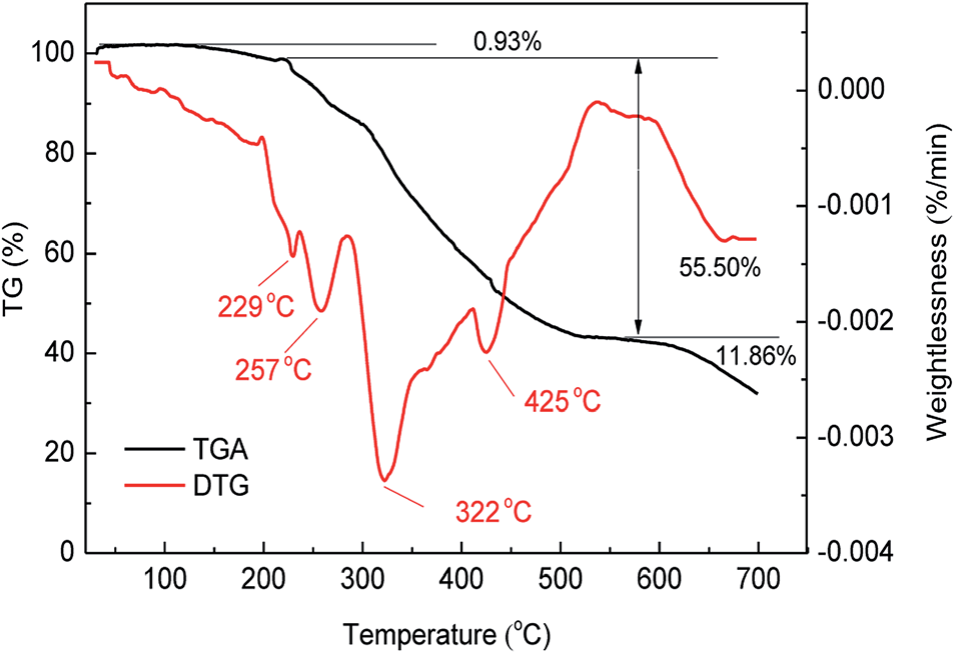
TGA and DTG of MAS-1.

The decomposition temperature of the polymer mostly depends on the thermal stability of the groups of macromolecules. Fig. 4 shows the thermogravimetric analysis (TGA) and differential thermogravimetry (DTG) curves for MAS-1. It can be seen that when the weight loss rate is 50%, the decomposition temperature is 454 °C, indicating the good thermal stability of MAS-1.^19^ The curves showed that MAS-1 had three stages of mass loss. The first stage was in the range of 25 °C to 217 °C with a mass loss of 0.93% due to the evaporation of intramolecular and intermolecular moisture. The second stage occurred in the temperature range of 217 °C to 579 °C with a mass loss of 55.50%. This stage could be associated with the pyrolysis temperature for the breakdown of C–N chemical bonds, the benzene and sulfonic acid groups. The final stage of the mass loss of MAS-1 appeared at above 579 °C, which can be attributed to carbonization. It should be emphasized that most of the mass loss of MAS-1 occurred well above 200 °C, indicating that MAS-1 is stable at 200 °C, suggesting that MAS-1 has better heat resistance and meets the requirement for a fracturing fluid at 200 °C.

### X-ray diffraction (XRD) of MAS-1

**Fig. 5:**
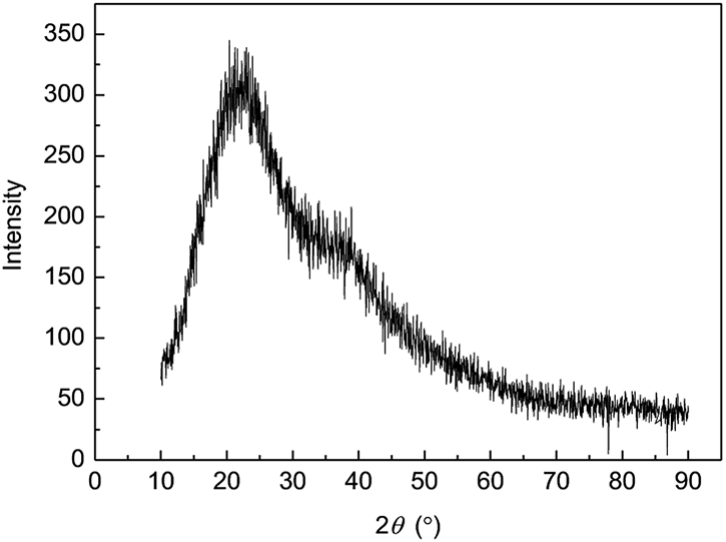
XRD of MAS-1.

X-ray diffraction pattern analysis is another way to understand element, internal atom or molecular structure and morphology of materials. The wide-angle XRD spectrogram of MAS-1 was shown in Fig. 5. It illustrated that MAS-1 is an amorphous structure because there were no sharp peaks rather than wide diffuse peaks. MAS-1 does not absorb outside energy as crystals to destroy their spatial lattice, which made it easily dissolvable in water.

### Water solubility of the copolymer

Generally, the conductance of a solution increases as a copolymer is dissolved in water continuously, and the conductivity of the solution is constant when the copolymer is dissolved completely. Therefore, a higher rate of increase in conductivity reflects a higher dissolution rate of a copolymer, which indicates that the water solubility of the polymer is better.

**Fig. 6:**
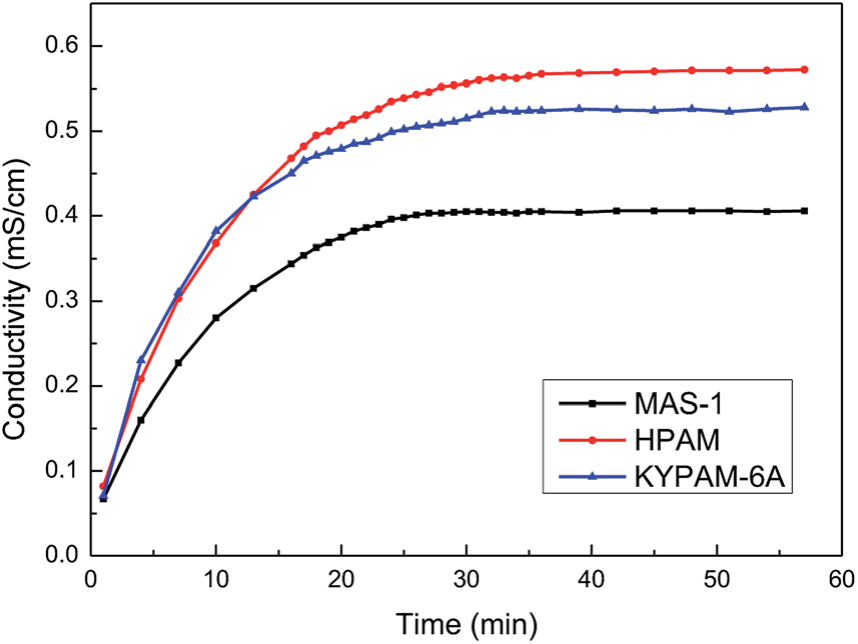
Curves of conductivity changes in the dissolution process of MAS-1.

Fig. 6 shows that the conductivity of the solution is small before the copolymer is added. As the polymer dissolves in water, the conductivity of the solution increases. Finally, the conductivity of the solution becomes constant, indicating that the copolymer dissolved completely. The dissolution times of MAS-1, hydrolyzed polyacrylamide (HPAM), and KYPAM-6A were 27 min, 39 min, and 33 min, respectively, which indicates that the water solubility of MAS-1 is better than that of HAPM or KYPAM-6A. There are two reasons for these experimental results. The molecular weight of MAS-1 is less than that of HPAM and KYPAM-6A, and the hydrophilic groups, such as sulfonate and carboxylate, in MAS-1 make the solubility of MAS-1 better than HPAM.^20^ The fast-dissolving property of MAS-1 is conducive to its preparation and application in hydraulic fracturing.

### Shear resistance

**Fig. 7:**
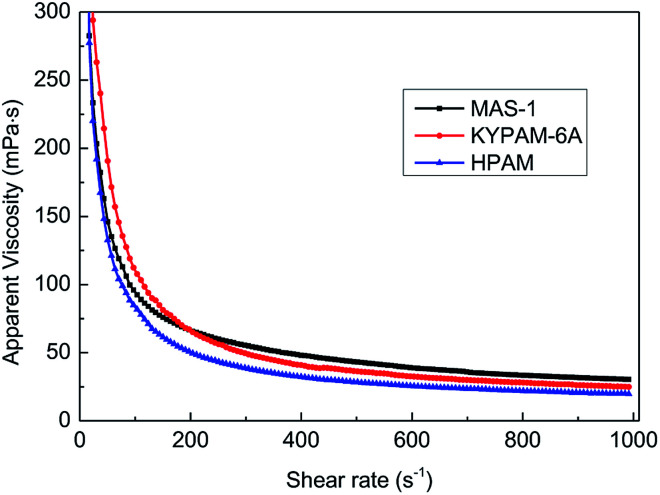
Effect of the shear rate on the apparent viscosity of the polymer solution.

In hydraulic fracturing, fracturing fluids are subjected to greatly varying shear rates as they move from the ground pump sets to the target reservoir through pipelines. Excellent shear-resistance ensures that the fracturing fluid will maintain good viscosity and proppant suspension. Viscosity is usually used to evaluate the shear resistance of the fracturing fluids under variable shear conditions. Fig. 7 shows the results of the continuously variable shear of MAS-1, KYPAM-6A, and HPAM. It is notable that the viscosity of MAS-1 decreased more slowly than the viscosity of KYPAM-6A and HPAM as the shear rate continuously increased. At 1000 s^−1^, the retention rates of the viscosity of MAS-1, KYPAM-6A, and HPAM were 56.2%, 46.3%, and 45.8%, respectively. The results of the variable shear measurement demonstrated that because the bending of the polymer backbone was prevented by rigid groups introduced in MAS-1, it has a better shear-resistance than KYPAM-6A or HPAM. Therefore, the viscosity retention rate of MAS-1 was relatively higher than that of KYPAM-6A or HPAM. Furthermore, the variable shear property of the solution (0.3 wt% MAS-1) was fitted by three physical models (Carreau, Carreau-Yasuda, Ostwald de Waele), which are shown in eqn (1)–(3).^21–23^

Carreau:1
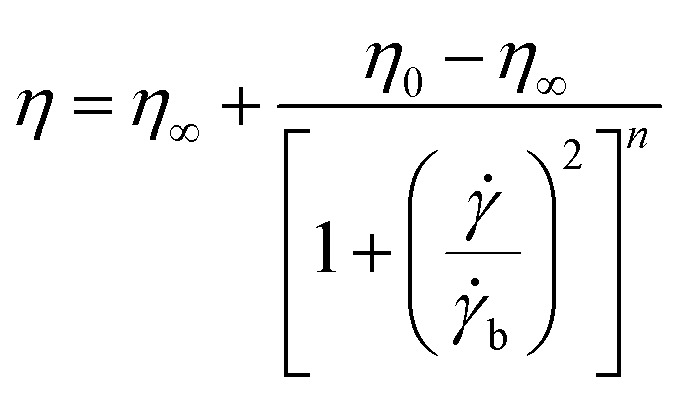


Carreau-Yasuda:2
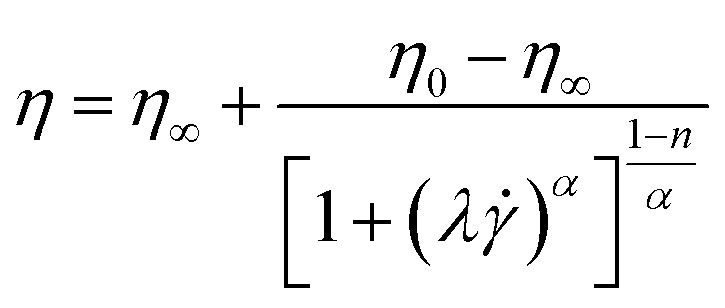


Ostwald de Waele:

3 *η* = *K

<svg xmlns="http://www.w3.org/2000/svg" version="1.0" width="10.615385pt" height="16.000000pt" viewBox="0 0 10.615385 16.000000" preserveAspectRatio="xMidYMid meet"><metadata>
Created by potrace 1.16, written by Peter Selinger 2001-2019
</metadata><g transform="translate(1.000000,15.000000) scale(0.013462,-0.013462)" fill="currentColor" stroke="none"><path d="M320 960 l0 -80 80 0 80 0 0 80 0 80 -80 0 -80 0 0 -80z M160 760 l0 -40 -40 0 -40 0 0 -40 0 -40 40 0 40 0 0 40 0 40 40 0 40 0 0 -280 0 -280 -40 0 -40 0 0 -80 0 -80 40 0 40 0 0 80 0 80 40 0 40 0 0 80 0 80 40 0 40 0 0 40 0 40 40 0 40 0 0 80 0 80 40 0 40 0 0 120 0 120 -40 0 -40 0 0 -120 0 -120 -40 0 -40 0 0 -80 0 -80 -40 0 -40 0 0 200 0 200 -80 0 -80 0 0 -40z"/></g></svg>

*^*n*−1^

where *η*_0_ represents the zero shear viscosity (Pa s), *η*_∞_ represents the ultimate shear viscosity (Pa s), *γ̇*_b_ represents the shear rate parameters, *n* represents the flow behavior index, *λ* represents the relaxation time, *K* represents the consistency coefficient (mPa sn), and *α* represents the Carreau constant.

**Fig. 8:**
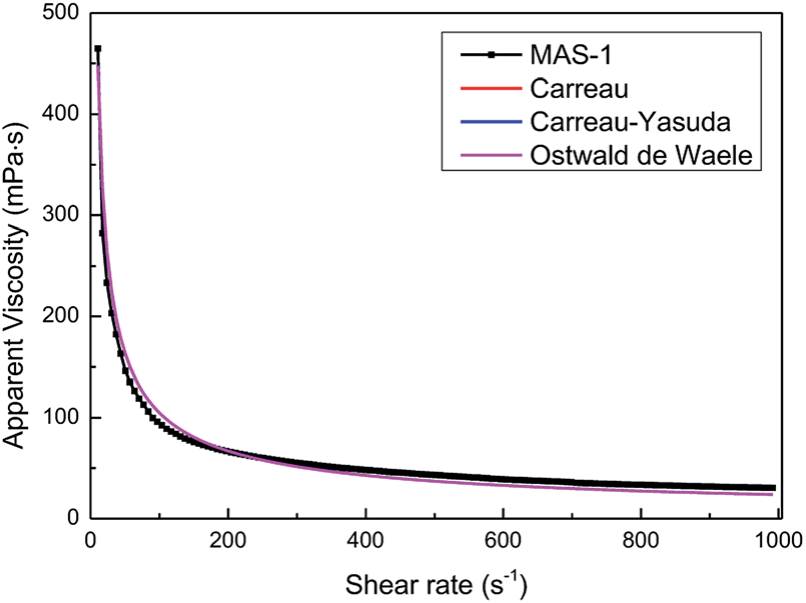
Fitting curves of the three models.

Fig. 8 and Table 1 show the fitting results and parameters. All fitting curves coincided with the experimental data (*r* = 0.9931). Therefore, the fitting parameters can reflect the actual variable shear property of the solution. In addition, wider cracks in the fracturing process can be formed when the non-Newtonian fluid (0.3 wt% MAS-1) is used to suspend the proppant because the flow behavior index is smaller (*n* < 1) and the consistency coefficient is larger (*K* = 2049).

**Table 1:** Fitting parameters of the three models

Fitting model	Fitting parameters	Fitting results	Correlation value (r)
Carreau	*η*_0_	10 740	0.9931
	*η*_∞_	0	
	*γ*_b_	0.07677	
	*n*	0.3227	
Carreau-Yasuda	*η*_0_	13 730	0.9931
	*η*_∞_	0	
	*λ*	−19.06	
	*n*	0.3547	
	*α*	2	
Ostwald de Waele	*K*	2049	0.9931
	*n*	0.3547	

### Rheological property measurement

**Fig. 9:**
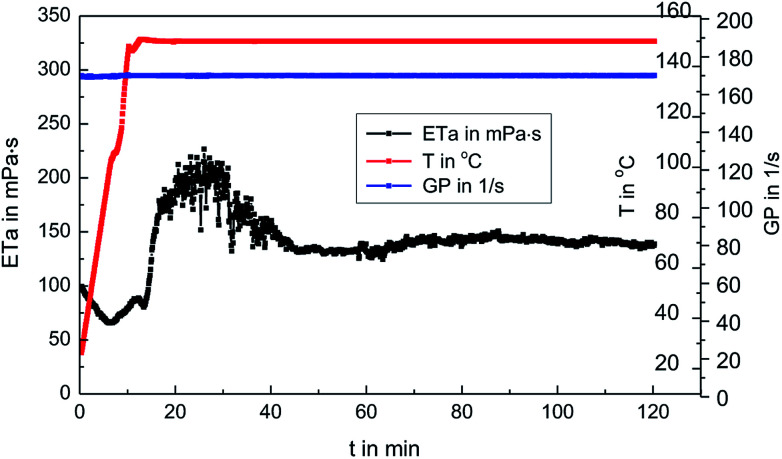
Rheological properties of 0.3 wt% MAS-1 with 0.8 wt% Zr-CL fracturing fluid at 150 °C, 170 s^−1^.

**Fig. 10:**
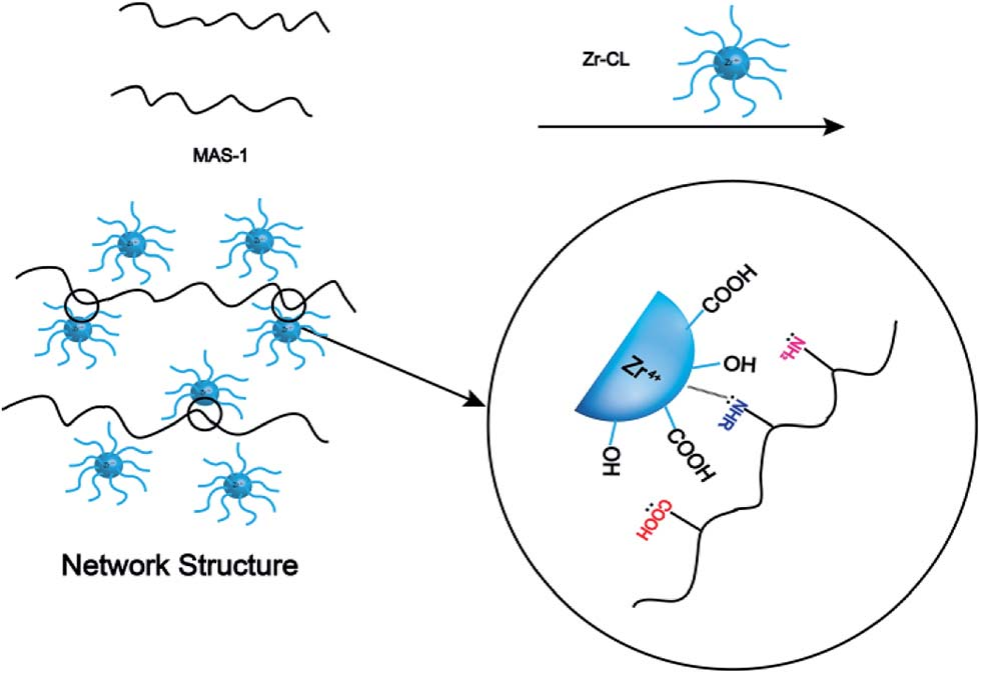
Crosslinking mechanism diagram of MAS-1 & Zr-CL.

The fracturing fluid was prepared by mixing 0.3 wt% MAS-1 and 0.8 wt% Zr-CL. Then, fracturing fluid was placed into the RS600 rheometer at 150 °C and 170 s^−1^. Fig. 9 shows that with thermal shear, the viscosity of the fracturing fluid generally decreased at first and then increased. Later, the viscosity dropped again and eventually remained around 135 mPa s. This may be due to the reaction of Zr-CL with the MAS-1 in aqueous solution. We concluded that the weaker bonds, such as hydrogen bonds, in the polymer solution are destroyed during thermal shearing. At the same time, the chelating agents on the zirconium atoms gradually fall off, so the empty orbitals on the zirconium atoms are gradually exposed to the lone-pair electrons on the oxygen and nitrogen atoms in the MAS-1.^24^Fig. 10 shows the crosslinking mechanism for MAS-1 and Zr-CL. There are some chelating bonds forming between the zirconium atoms and MAS-1. However, the chelating bonds are weaker than covalent bonds, so they are more easily broken by the thermal shear. From the macro point of view, Fig. 9 shows that the viscosity of the fracturing fluid decreases first, then increases, decreases again, and then stabilizes at about 135 mPa s after 120 min. Therefore, the fracturing fluid can maintain excellent thermo-shear stability at 150 °C. In addition, it should be emphasized that the initial viscosity of the fracturing fluid is less than 100 mPa s, which is significant for fracking, especially in deep or ultra-deep wells, because there would be less frictional resistance in the pumping process.

### Viscoelasticity

To a certain extent, the elasticity of the fracturing fluid reflects the density of the network structure in the solution. A stronger spatial network structure will result in a larger storage modulus, indicating more extensive network structures in fracturing fluids.

**Fig. 11:**
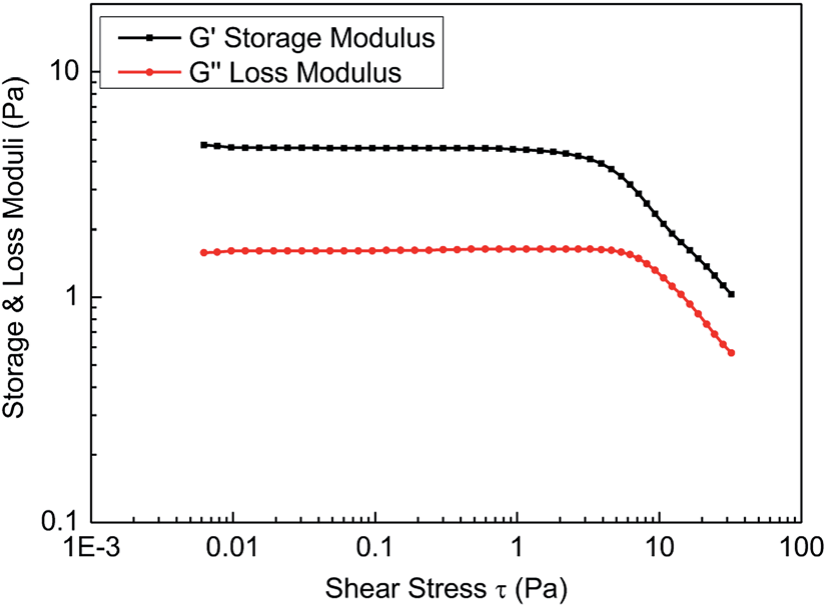
The stress scanning curve of the fracturing fluid.

Shear stress must be within the linear viscoelastic area during material property constant measurements, so linear viscoelastic zone measurements of the fracturing fluids should be conducted prior to the viscoelasticity measurements. Fig. 11 shows the results that were obtained through stress scanning experiments with a frequency of 1 Hz. The results show that the change in the storage modulus (*G*′) and loss modulus (*G*′′) of the fracturing fluid was small and that the ratio of storage modulus and loss modulus is 2.842 when the shear stress was less than 3.0 Pa, which indicates that the solution is in the linear viscoelastic zone. Therefore, the shear stress should be less than 3.0 Pa during the frequency scanning experiment for the fracturing fluids. For experimental convenience, frequency scanning was usually performed when the shear stress was set to 1.0 Pa.

**Fig. 12:**
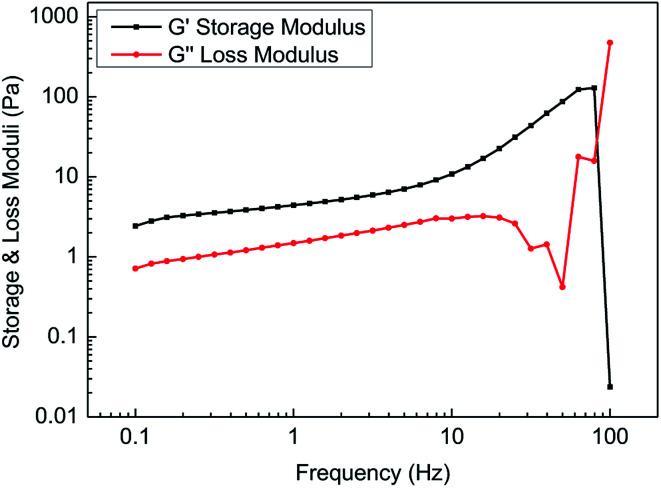
The frequency scanning curve of the fracturing fluid.

Fig. 12 shows the variation in the storage modulus and loss modulus with frequencies ranging from 0.1 Hz to 100 Hz. It is found that the storage modulus was always greater than the loss modulus with increasing scanning frequency in the linear viscoelastic zone. However, in the non-linear viscoelastic zone, the mutual entanglement among the molecular chains was destroyed, and most of the energy became thermal energy. Therefore, it is unnecessary to research the viscoelasticity of the fracturing fluids in the non-linear viscoelastic zone.

### Microstructure analysis

**Fig. 13:**
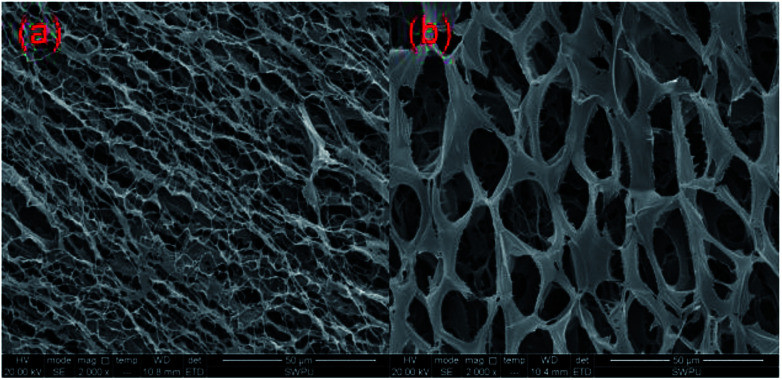
SEM images of the aggregation state of the different fluids. (a) 0.3 wt% MAS-1, (b) 0.3 wt% MAS-1 + 0.8 wt% Zr-CL.

It is well accepted that the macroscopic properties of the copolymers are determined by their microstructures. The change of the storage modulus and loss modulus of the MAS-1 fracturing fluids implies that their spatial network structure (SNS) has experienced microstructural changes. Fig. 13a and b show the SNS of the MAS-1 solution without and with the crosslinker. It is observed that the SNS of the MAS-1 solution with the crosslinker was denser than the solution without the crosslinker. This can be attributed to the chelation of the polymer with the crosslinker that makes the SNS denser than the solution without the crosslinker. From a macro point of view, the viscoelasticity and thermal stability of the polymer solution improved sharply, indicating that the MAS-1 fracturing fluid is an efficient carrier of the proppant into the cracks.

### Proppant carrying capacity (PCC)

**Fig. 14:**
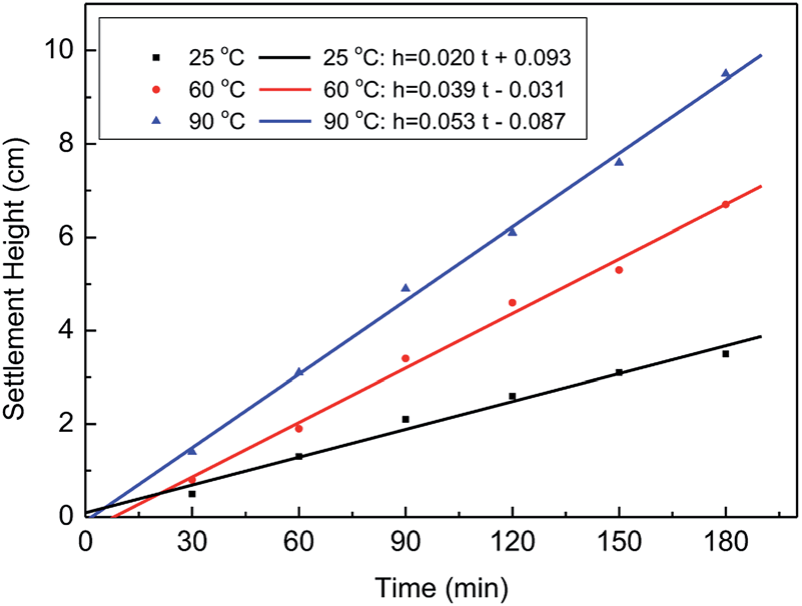
Static settlement curve of the fracturing fluid at different temperatures.

To determine the proppant carrying capacity, fracturing fluid was poured into a 50 mL cylinder with proppant (20/40 mesh), and the settlement height and time were measured at 25 °C, 60 °C, and 90 °C. Then, the settlement height and time were used to calculate the sedimentation velocities. Fig. 14 and Table 2 show that the sedimentation velocities of the proppant were 0.0194 cm min^−1^ at 25 °C, 0.0372 cm min^−1^ at 60 °C, and 0.0528 cm min^−1^ at 90 °C. It is clear that the sedimentation velocities of the proppant at three different temperatures were all less than 1.08 cm min^−1^, indicating that the fracturing fluid can meet the requirement of proppant suspension and transportation in fracking.^25^

**Table 2:** Static settlement velocities of the fracturing fluid at different temperatures

Temperature (°C)	Time (min)	Height (cm)	Sedimentation rate (cm min^−1^)
25	180	3.5	0.0194
60	180	6.7	0.0372
90	180	9.5	0.0528

### Gel breaking evaluation

The viscosities of the fracturing fluid with different concentrations of breaker were investigated at 90 °C. Table 3 shows these results. The results show that the fracturing fluid was broken thoroughly within 3 h when amounts of 0.10 wt% APS were added. The broken fluid is transparent without any visible residues.

**Table 3:** The preferred amount of APS

Breaking time (h)	Viscosity (mPa s)			
	0.02 wt% APS	0.04 wt% APS	0.08 wt% APS	0.10 wt% APS
0.5	180	162	138	123
1.0	168	147	108	84
1.5	141	126	81	66
2.0	126	111	54	42
2.5	108	93	36	21
3.0	87	72	18	4.5

### Core damage

The core damage tests were measured by injecting the broken fluid into the core at 25 °C, 60 °C, and 90 °C to further detect the damage of the fracturing fluid to the formation. Table 4 shows the experimental results. The results show that the permeability damage rate was between 12.64% and 17.62%. That is to say, the damage rate of this fluid is less than the industry standard (≤30%), which indicates that the fracturing fluid does little damage to the reservoir.

**Table 4:** Experimental results of core damage

Temperature (°C)	Core parameters		Core permeability (mD)		Permeability damage rate (%)
	Diameter (cm)	Length (cm)	*K*_1_	*K*_2_	
25	2.50	4.25	4.36	3.81	12.64
60	2.48	4.64	4.54	3.74	17.62
90	2.50	4.50	3.65	3.08	15.7

### Field-scale friction reduction evaluation

**Fig. 15:**
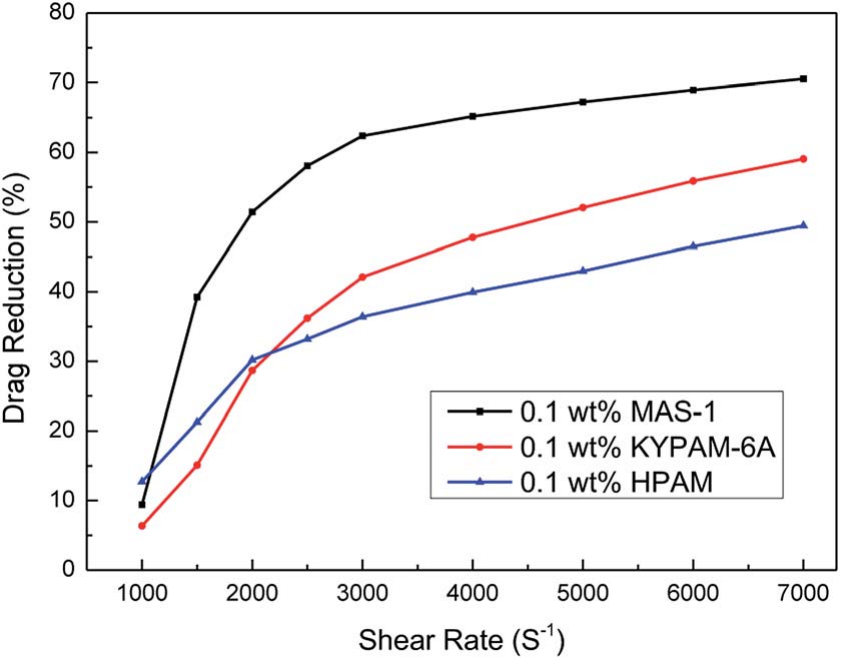
The variation of drag reduction rate with shear rate.

Fig. 15 shows the relationship between the shear rate and drag reduction rate for fluids with MAS-1, KYPAM-6A, and HPAM. It is clear that the drag reduction rates of fluids with MAS-1, KYPAM-6A, and HPAM increase with increasing shear rate. It is concluded that polymers form an elastic bottom layer near the inner wall of the pipeline. The elastic bottom layer thickens gradually as the shear rate increases, indicating that the drag reduction rate is increasing.^26^ Meanwhile, the drag reduction rate reaches a maximum value when the thickness of the elastic bottom layer reaches its limit at the center of the pipe. Moreover, it should be emphasized that the drag reduction rate of MAS-1 fluids was always higher than for KYPAM-6A or HPAM fluids, which were up to 70.5%, indicating that the MAS-1 solution could reduce friction and improve the pump efficiency in fracking. Therefore, this fluid is an alternative to presently used fracturing fluids for low permeability reservoir stimulation.

## Conclusions

In order to obtain a high-performance fracturing fluid, a new terpolymer (MAS-1) was synthesized by free-radical copolymerization under optimized conditions using the single variable method. MAS-1 was characterized by FT-IR. Investigations of the use of this polymer in the fracturing fluid were carried out, and the following conclusions were made:

(1) TGA suggested that MAS-1 has better heat resistance and meets the requirement for a fracturing fluid at 200 °C. XRD and conductivity tests showed that MAS-1 has good water solubility. In addition, it was investigated that MAS-1 fracturing fluid maintained a viscosity of about 135 mPa s after 120 min at 170 s^−1^ and 150 °C. (2) Viscoelasticity determination and SEM images showed that there was a dense and robust spatial network structure in the MAS-1 fracturing fluid. The sedimentation velocity of proppant was 0.0528 cm min^−1^ at 90 °C. (3) When approximately 0.10 wt% ammonium persulfate was added, the viscosity of the broken fluid was 4.5 mPa s at 90 °C after 3 h. The permeability damage rate of the gel broken fluid to cores was 12.64% to 17.62%, indicating that the fracturing fluid causes little damage to the reservoir. (4) The drag reduction rate of MAS-1 solutions was always higher than for KYPAM-6A or HPAM solutions, with a shear rate ranging from 1000 s^−1^ to 7000 s^−1^, which would reduce the pump pressure and increase the efficiency of fracking.

## Conflicts of interest

There are no conflicts to declare.

## Supplementary Material

RA-009-C8RA09483G-s001
