# Supplementary material for: Preparation of a novel fracturing fluid with good heat and shear resistance
Source: RSC Adv. 2019 Jan 9;9(3):1199–207. doi: 10.1039/c8ra09483g (PMC9059618; doi:10.1039/c8ra09483g)
Supplement: RA-009-C8RA09483G-s001 [file RA-009-C8RA09483G-s001.pdf]

Electronic Supplementary Material (ESI) for RSC Advances.
This journal is © The Royal Society of Chemistry 2018

Electronic Supplementary Material (ESI) for RSC Advances. This journal is © The Royal Society of Chemistry 2018

Supporting Information for

Preparation of a Novel Fracturing Fluid with Good Heat and Shear Resistance

Yang Zhang, Jincheng Mao *, Tao Xu, Zhaoyang Zhang *, Bo Yang, Jinhua Mao, and Xiaojiang Yang*

State Key Laboratory of Oil and Gas Reservoir Geology and Exploitation, Southwest Petroleum University, Chengdu 610500, P. R. China

Experimental Section

Characterization. The purified MAS-1 with potassium bromide(KBr) tablet was prepared to characterize its molecular structure. The infrared spectroscopy (NICOLET 6700, USA) had a wave number range between 4000 cm^{-1} and 400 cm^{-1}, and a resolution of 0.01 cm^{-1}. The nuclear magnetic resonance hydrogen spectrum (^1H NMR) of copolymers in D_2O were measured using Bruker AVANCE III HD 400 (Bruker, Switzerland). The concentration of the polymer solution was 100 mg/L.

Thermogravimetric Analysis (TGA). TGA of MAS-1 was measured by Thermo Gravimetric Analyzer (DSC 823 TGA/SDTA85/e). In this test, the protective gas and the purge gas were both 99.999% of nitrogen with 50 mL/min. The sample was heated from 30 ^oC to 700 ^oC with a heating rate of 10 ^oC/min.

X-ray Diffractometry (XRD). The MAS-1 was measured by X-ray diffraction measurements using an X-ray diffractometer with Cu, k_α radiation target at 40 mA and 40 kV and a scan rate of 1 deg/min, step size of 0.05 degree, with the scattering angle (2θ) ranging from 10° to 90°.

Water Solubility Test. In this experiment, the water solubility of the copolymers was obtained by conductivity method. The solution conductivity was measured by using a DDS-307$^+$ conductivity meter (Chengdu century Ark Technology Co., Ltd.). Copolymer particle samples were dispersed

and dissolved in deionized water performing at 25 °C. The water solubility curves were obtained using the dissolution time, which is defined as the time from initially adding the polymer to the solution conductivity stabilizing.

Viscoelasticity. The viscoelasticity as a function of stress and frequency sweep for 0.3 wt% MAS-1 solution was measured using an Anton PPar rheometer(MR302) with CP50-1-SN30644 plate fixture (diameter=0.099 mm). To ensure the consistency of the experimental conditions, all samples were measured at 25 °C.

Microstructure Analysis. The morphology was investigated using an environmental scanning electron microscope (ESEM, Quanta 450, USA). The systems of 0.3 wt% MAS-1 solution without and with crosslinker were prepared and dropped on glass slides to obtain samples for ESEM observation, respectively. All of samples were frozen using liquid nitrogen. The frozen surfaces of the samples were observed with ESEM operating at an accelerating voltage of 20 KV.

Core Damage. The core damage was evaluated with a core-flow test. The experimental setup was shown in Figure 1. The core was first vacuumed and saturated with standard salt water (2.0% KCl + 5.5% NaCl + 0.45% $MgCl_2$ + 0.55% $CaCl_2$). The experiment was performed at 25 °C, 60 °C and 90 °C. The core was established with standard salt water by injection in the forward direction. The permeability was obtained, named as K_1. Then broken fluid was injected into the core in the reverse direction for 2 hours. The standard salt water was injected into the core in the forward direction at a steady flow rate in order to obtain the retained permeability, named as K_2. Finally, the rate of permeability damage (η_d) was calculated by the equation (1). It should be emphasized that the standard salt water and broken fluid were injected into the core by the nitrogen. And the core was held and pressed by the confining pressure.

$$\eta_d = \frac{K_1 - K_2}{K_1} \times 100\% \qquad (1)$$

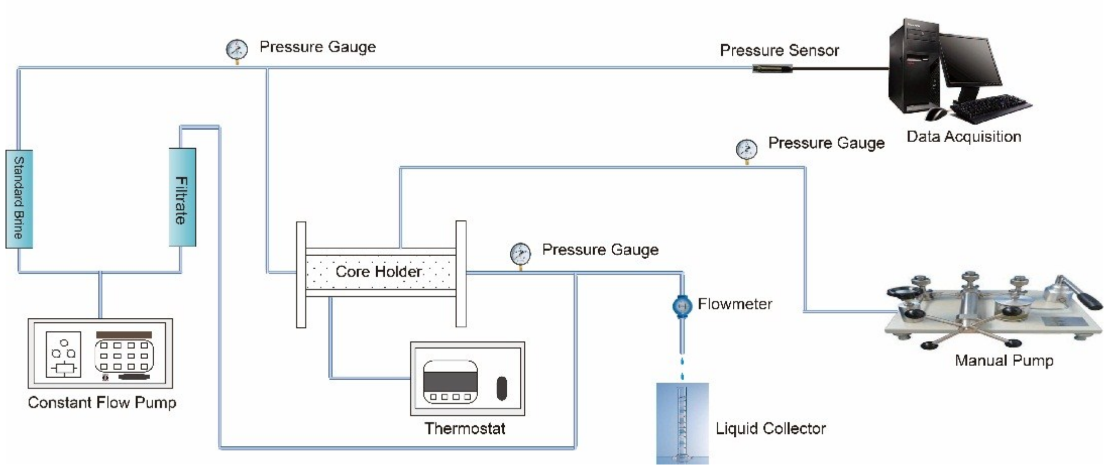

Figure 1. The setup of core damage test.

Friction Reduction Testing Procedure. The drag reduction rate of drag reducing agent, such as MAS-1, HPAM and KYPAM-6A, was measured by self-made pipeline loop friction system (D=6 mm, L=4000 mm). It was recorded that the pressure difference of the clear water and the drag reducing agent solution go through the pipeline loop under different flows. The drag reduction (DR) was calculated by the equation (2).

$$\eta = \frac{\Delta P_0 - \Delta P_1}{\Delta P_0} \times 100\% \tag{2}$$

where η represents the drag reduction of MAS-1, ΔP_0 represents the pressure drop of clean water (MPa), ΔP_1 represents the pressure drop of MAS-1 (MPa).
